# Behavioral evidence for the origin of Chinese Kunming dog

**DOI:** 10.1093/cz/zoaa081

**Published:** 2021-01-12

**Authors:** Jin-Xiu Li, Qing-Guo Huang, Shi-Zhi Wang, Qi-Jun Zhou, Xu Gao, Ya-Ping Zhang, Guo-Dong Wang

**Affiliations:** 1 State Key Laboratory of Genetic Resources and Evolution, Kunming Institute of Zoology, Chinese Academy of Sciences, Kunming, 650223, China; 2 Kunming Police Dog Base of the Chinese Ministry of Public Security, Kunming, 650204, China; 3 Harbin Police Dog Training Centre, Heilongjiang General Station of Exit and Entry Frontier Inspection, Harbin, 150000, China; 4 Center for Excellence in Animal Evolution and Genetics, Chinese Academy of Sciences, Kunming, 650223, China

**Keywords:** behavior test, Chinese Kunming dog, origin

As the only working dog independently developed and bred in China, Chinese Kunming dog (CKD) was approved as a new breed by the Chinese National Livestock and Poultry Genetics Commission in 2007. Now CKD is widely used by the Chinese army and police and has been exported to Singapore, Thailand, Vietnam, North Korea, and other more than 10 countries as working dog. Previously published studies of CKD have focused on coat color genetics (Wang et al. 2013) and reproduction performance (Wei et al. 2018). The study by Wang et al. (2013) pointed out that the breed of CKD was developed in the 1950s by hybridizing German shepherd dogs (GSDs) imported from the Soviet Union with Kunming indigenous village dogs. Inspired by the confirmed genetic basis of breed-based behavioral stereotypes and the existed heritability of behavioral traits estimated in Swedish Army dogs (van der Waaij et al. 2008, Arvelius et al. 2014), a hypothesis was proposed that CKD might have a similar behavioral stereotype to its original breed. However, there are no studies to analyze and compare the behavior of CKD and GSDs. Hence, referring to the PAWS (http://landofpuregold.com/PAWS.htm) and the GSD test (Ruefenacht et al. 2002), a general working dog behavior test endorsed by the Chinese Ministry of Public Security (described in Supplementary Table S1) was conducted for 262 breeding dogs kept in the Kunming Policy Dog Base.

There are 4 breeds among the 262 breeding dogs including Belgian Malinois dog (BMD, *N = *117), CKD (*N = *115), Eastern German Shepherd dog (EGSD, *N = *7), and GSDs (*N = *23) in the Kunming Police Dog Base of the Chinese Ministry of Public Security (described in Supplementary Table S2). The reliability index Cronbach’s α coefficient of the test method was 0.672–0.863, and the overall scheme reached 0.792 and correlation’s α coefficient between predictive validity and test items was 0.273–0.855 with *P* < 0.05, which confirmed the reliability and validity of the behavioral test. The statistics of number, age, and gender of those 4 breeds used in the test also is shown in Supplementary Table S2. In the test, the following behavior items were scored: following (FO), stranger reaction (SR), gunshot response (GR), stability test (ST), fetch test (FT), tug of war (TW), and attention item (AI), (Supplementary Table S1). Scores for each item in the test were recorded ranging from 1 to 5, with 1 having the worst performance and 5 having the best performance, and the average scores (with standard deviation) of behavior test for the 4 breeds are shown in Supplementary Table S2.

Multiway analysis of variance (ANOVA) was performed to explore if individual test scores were affected by the factors of breeds, sex, age of year, and their interactions. The multiway ANOVA results (Supplementary Table S3) showed that the factor of breeds, sex significantly affected individual test scores of all the behavioral items, and age of year also significantly affected individual test scores of all the behavioral items except for the GR. Besides, the interaction between the factors breed and sex significantly affected the individual test scores of GR (*P *=* *0.009) and ST (*P* = 0.038), and the interaction between factors breed, age, and sex significantly affected the individual test scores of FT (*P* = 0.024). Hence, a generalized linear model (GLM) assuming under Gaussian distribution was performed to estimate breed effects on each test items. Only the factors breed, age of year, and sex shown from the results of previous ANOVA were used to make the GLM models for each item, so that the breed effect could be estimated consistently across behavioral items. The estimated effects of factors breed, age of year, and sex for the 7 behavioral items are shown in Supplementary Table S4, and the values of intercept plus breed effects were graphed in Figure 1A. As shown in Figure 1A, the CKD had the lowest breed effects in all the behavioral items and the EGSD had the highest breed effects in all the behavioral items. Besides, the estimated breed effect GR of the GSD (3.78) was close to that of the ESGD (3.92), and the estimated breed effect TW of the BMD (3.16) was very close to that of the ESGD (3.20). It was observed that the breed-effect pattern on behavioral items of CKD is generally similar to that of GSD without considering the absolute value of the breed effects (Figure 1A). To quantify the similarity of overall behavioral stereotypes between CKD and other breeds, Euclidean distance was calculated by hierarchical cluster analysis using the previously estimated breed effects of the 7 items together for the 4 breeds. The cluster dendrogram of behavioral stereotype based on estimated breed effects are shown in Figure 1B and the GSD was the mostly close to the CKD in the aspect of behavior (the Euclidean distance is 1.26). Besides, the Euclidean distance between CKD and BMD and EGSD were 1.63 and 2.65 and more details about Euclidean distance among the 4 breeds could be check-in Supplementary Table S5.

**Figure 1.**
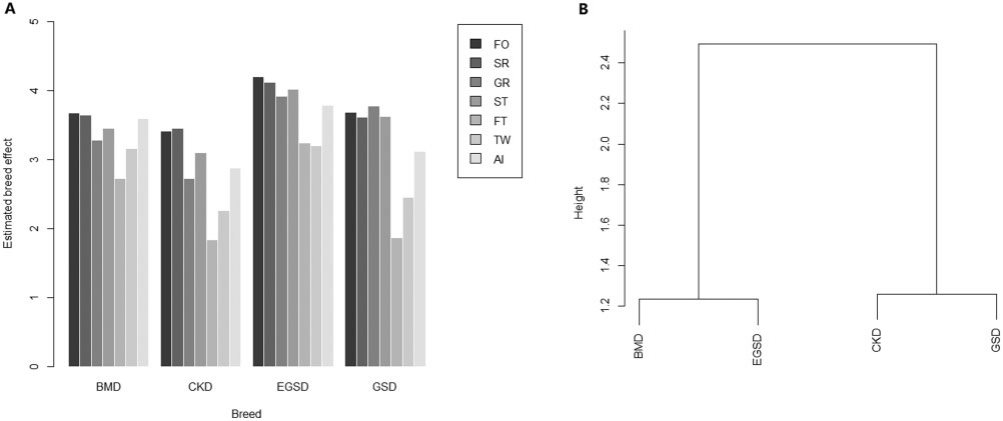
Bar graph of estimated breed effect of behavioral test items by breeds (**A**) and cluster dendrogram of behavioral stereotype based on estimated breed effects (**B**).

In summary, we estimated the breed effects on 7 behavioral test items that used to evaluate the general performance and capability of breeding dogs in Policy Dog Base. The study demonstrated the behavioral stereotypes in working dogs are varied from breed to breed, which is similar to what we have observed that dogs as a species have evolved with an extraordinary level of behavioral diversity across breed (Ostrander et al. 2019). Besides, the evidence from the comparison of behavioral stereotype implied CKD might origin from the hybrid between Kunming indigenous village dogs and GSD instead of EGSD, which could be confirmed if genome sequencing data of the breeds were comparatively analyzed in the future study. Furthermore, the overall behavioral test scores of CKD are lower than the other 3 breeds as working dog, which might suggestive that more intensive selection on evaluated performance behavior and temperament of CKD is needed.

## Supplementary Material

zoaa081_Supplementary_Data
