# Supplementary material for: Behavioral evidence for the origin of Chinese Kunming dog
Source: Curr Zool. 2021 Jan 12;67(4):469–71. doi: 10.1093/cz/zoaa081 (PMC8489011; doi:10.1093/cz/zoaa081)
Supplement: zoaa081_Supplementary_Data [file zoaa081_supplementary_data.zip › supplementary_materials.doc]

***Letter to the Editor***

**Behavioral evidence for the origin of Chinese Kunming dog**

Jin-Xiu Li^a, #^, Qing-Guo Huang^b^^,#^, Shi-Zhi Wang^a^, Qi-Jun Zhou^a^, Xu Gao^d^, Ya-Ping Zhang^a, c,^ *, Guo-Dong Wang^a, c,^ *

^a^State Key Laboratory of Genetic Resources and Evolution, Kunming Institute of Zoology, Chinese Academy of Sciences, Kunming 650223, China; ^b^Kunming Police Dog Base of the Chinese Ministry of Public Security, Kunming 650204, China; ^c^Center for Excellence in Animal Evolution and Genetics, Chinese Academy of Sciences, Kunming 650223, China; ^d^Harbin Police Dog Training Centre, Heilongjiang General Station of Exit and Entry Frontier Inspection, Harbin 150000, China.

*Address correspondence to Ya-Ping Zhang, Email: [zhangyp@mail.kiz.ac.cn](mailto:zhangyp@mail.kiz.ac.cn), Guo-Dong Wang, Email: [wanggd@mail.kiz.ac.cn](mailto:wanggd@mail.kiz.ac.cn).

^#^These authors contributed equally

Handling editor: Jian-Xu Zhang,

Received on 14 October 2020; accepted on 124 December 2020

**Supplementary Methods and objects**

**Behavioral test items**

Based on the test content of PAWS (<http://landofpuregold.com/PAWS.htm>) and the study of Ruefenacht et al. (2002) on German Shepherd dogs, following (FO), stranger reaction (SR), gunshot response (GR), stability test (ST), fetch test (FT), tug of war (TW), and attention item (AI) test items were identified as shown in Supplementary Table 1.

**Experimenters**

The experimenters consist of testers, graders, trainers, and strangers. The tester was a stranger to the dogs and was responsible for the implementation of the test, the grader was responsible for observing the dog’s behavioral response and providing the results against the scoring criteria and recording the behavioral response, the trainer was responsible for assisting the tester, and stranger was unfamiliar to the dogs. Testers and graders were familiar with the testing process and methods, and trainers and other personnel were familiar with relevant testing procedures.

**The test area**

The test area was an open space with an area of no less than 50m^2^, 200 meters away from the test kennel. The environment was relatively quiet, with no interference from other animals or people.

**The procedure**

The test time was from 3:00 PM to 5:00 PM before feeding. Dogs were tested separately. Before the test, they were familiar with the environment for 3-5min. The test contents were completed according to the test items in Table S1. For each one, only the first behavioral response of the tested dog was recorded. One test lasted for 1 minute, and it took about 15 minutes to complete all the test items. According to the five-point scale, the score was divided into 5 levels, 1-1.9, 2-2.9, 3-3.9, 4-4.9 and 5 for each test item, with 1 having the worst performance and 5 having the best performance. The total score of the seven items was 35. The score was recorded according to the standard set by the behavioral response control, and the behavioral response was recorded if the score was beyond the range of the score. The scores of each test were calculated by averaging the scores of all three graders.

**Objects**

The dogs involved in the study came from the Kunming Police Dog Base of the Chinese Ministry of Public Security. Four dog breeds participating in the behavior test were Belgian Malinois dog, Chinese Kunming dog, East German Shepherd, and German shepherd dog (Supplementary Table 2). At the onset of the behavioral test, the dogs had not been involved in any research study for at least 2 weeks. The test group ranged in age from 2 to 9 years.

**Statistical analyses**

The reliability and validity of the behavioral test were calculated using Statistics Analysis System (SAS) 6.12 (Littell et al. 1998) to evaluate the reliability and validity of the test methods.

**Supplementary Tables**

**Supplementary Table 1:** The behavioral test method for dogs.

| **Test contents** | **Test Methods** | **Rating basis** | **Score** |
| --- | --- | --- | --- |
| Following（FO） | The dog owner brings the dog to the test field, and after 3-5 minutes of acclimation, the handler calls the dog to follow the dog takes off the rope. | Take the initiative and follow the trainer closely. the tail cocked; intermittently nibble at trouser legs or with a cheerful bark. | 5 |
|  |  | Take the initiative and follow the trainer immediately; the tail cocked; at the edge of the leg. | 4 |
|  |  | Take the initiative to follow the trainers walk; the tail hangs down; keep a distance from the trainer. | 3 |
|  |  | Hesitates to follow the handler to walk; the tail hangs down; keep a distance from the trainer. | 2 |
|  |  | Not responding or following trainers at all; just walk away. | 1 |
| Stranger reaction（SR） | The 4 strangers form 2 columns, 1.5m apart, and the trainer takes off the rope and leads the dog to pass through the 4 people. | The tail cocked; walk through the crowd of strangers with light and confident gait. | 5 |
|  |  | The tail cocked; carefully sniff through the crowd of strangers. | 4 |
|  |  | The tail hangs down; look around carefully to complete the stranger crowd shuttle. | 3 |
|  |  | Clipped tail, look around carefully to complete the stranger crowd shuttle. | 2 |
|  |  | Clipped tail, afraid to flinch and cannot complete the stranger crowd shuttle. | 1 |
| Gunshot response（GR） | Place the dog in the middle of the test field and the assistant, 10 meters from the dog, pulls the starting gun. | Ignore the sound; no curiosity; no effect on behavioral psychology or accompanied by barking. | 5 |
|  |  | Listen; be confident and calm; actively explore and identify the source of sound and bark. | 4 |
|  |  | Listen; stand still; actively identify the source of the sound; howl. | 3 |
|  |  | Listen; gradually retreat after standing still; passively identify the source of the sound and make a defensive bark. | 2 |
|  |  | Quickly wretched; retreat; hide. | 1 |
| Stability test（ST） | The trainer led the test dog, and the tester suddenly opened the automatic umbrella (1m in diameter) and randomly placed it on the ground when the dog noticed it at a distance of 1.5m. | Ignore the existence of the umbrella, confident and calm; the tail cocked; active aggressive barking. | 5 |
|  |  | Startled after watching umbrella bark; the tail cocked; or take the initiative to bite the umbrella with mouth. | 4 |
|  |  | Startled after watching umbrella bark; the tail cocked; dare not go near the umbrella. | 3 |
|  |  | Startled after staring at the umbrella hesitate to bark; the tail dropped and retreated intermittently. | 2 |
|  |  | Shocked; Clipped tail; Run away quickly. | 1 |
| Fetch test（FT） | The trainer uses a tennis ball to attract the dog's attention. When the dog notices the tennis, the trainer throws the ball 5-10 meters away from the dog and encourages the dog to take it. | Quickly chasing tennis to run back or play, like multiple round trips. | 5 |
|  |  | Quickly chasing tennis to run back or play, do not like multiple round trips. | 4 |
|  |  | Quickly chasing tennis to run back or play, go back to the trainer but don’t hand over the tennis ball. | 3 |
|  |  | Follow the direction of the tennis ball but run away, not back to the trainer. | 2 |
|  |  | The dog does not run to tennis and does not hold the tennis ball. | 1 |
| Tug of war（TW） | Use towel rods to tease the test dog to make it fully possessive. | When the dog gets the towel, the hind legs stay in place, and immediately drag and hold, or accompanied by foot picking and whining, for >60 seconds. | 5 |
|  |  | When the dog gets the towel, the hind legs stay in place, and immediately drag, do not encourage, or accompanied by foot picking and whining, for 30 to 60 seconds. | 4 |
|  |  | When the dog gets the towel, the hind legs stay in place, and immediately drag, do not encourage, or accompanied by foot picking and whining, for 15 to 30 seconds. | 3 |
|  |  | Repeatedly teasing and encouraging, just get it and put it down immediately. | 2 |
|  |  | Always do not bite; ignore it or just walk away. | 1 |
| Attention item（AI） | Trainers tease the dogs with items they are interested in, such as pom-poms, towel rods, etc. | Continue to follow the teasing object in a constant chase, or with a cheerful bark, for > 60 seconds. | 5 |
|  |  | Continue to follow the teasing object in a constant chase, or with a cheerful bark, for 30 to 60 seconds. | 4 |
|  |  | Continue to follow the teasing object in a constant chase, or with a cheerful bark, for 15 to 30 seconds. | 3 |
|  |  | Continue to follow the teasing object in a constant chase, or with a cheerful bark, for 0-15 seconds. | 2 |
|  |  | Ignore; no curiosity; no interest. | 1 |

Note: The trainer is a relatively fixed professional in the daily feeding management training of the test dog; the tester is the person who makes the test plan and is responsible for the scoring record, and taking the test for the first time and belonging to a stranger to the dog.

**Supplementary Table 2:** Information about working dogs taking the behavior test. Age data are presented as mean ± sd.

| **Subjects** | **Number**  **in test (N)** | **Age** | **Male (%)** | **FO** | **SR** | **GR** | **ST** | **FT** | **TW** | **AI** |
| --- | --- | --- | --- | --- | --- | --- | --- | --- | --- | --- |
| Belgian Malinois Dog (BMD) | 117 | 4.67±2.61 | 25.0 | 3.49±0.86 | 3.43±0.87 | 3.43±1.21 | 3.43±0.99 | 3.27±1.17 | 2.97±1.36 | 3.54±1.00 |
| Chinese Kunming Dog (CKD) | 114 | 4.66±2.31 | 41.4 | 3.35±0.85 | 3.40±0.84 | 3.03±1.07 | 3.22±0.92 | 2.60±1.05 | 2.36±1.22 | 3.00±1.02 |
| East German Shepherd Dog (EGSD) | 7 | 3.29±2.36 | 85.7 | 4.43±0.54 | 4.28±0.50 | 4.48±0.37 | 4.43±0.45 | 3.93±1.32 | 4.33±0.69 | 4.26±0.98 |
| German Shepherd Dog (GSD) | 23 | 4.00±1.62 | 52.2 | 3.67±0.75 | 3.54±0.76 | 4.07±0.79 | 3.76±0.73 | 2.85±1.03 | 2.45±1.08 | 3.26±0.85 |

Note: FO: Following; SR: Stranger reaction; GR: Gunshot response; ST: stability test; FT: Fetch test; TW: Tug of war; AI: Attention item.

**Supplementary Table 3:** Significance of factors affected the individual behavioral test scores by Multi-way ANOVA.

| **Factor** | **df** | **FO** | **SR** | **GR** | **ST** | **FT** | **TW** | **AI** |
| --- | --- | --- | --- | --- | --- | --- | --- | --- |
| Breed | 3 | **0.001** | **0.014** | **<0.000** | **<0.000** | **<0.000** | **<0.000** | **<0.000** |
| Age | 9 | **0.002** | **0.007** | 0.106 | **0.041** | **0.002** | **0.005** | **0.029** |
| Sex | 1 | **<0.000** | **<0.000** | **<0.000** | **<0.000** | **<0.000** | **<0.000** | **<0.000** |
| Breed*Age | 14 | 0.069 | 0.075 | 0.886 | 0.373 | 0.687 | 0.311 | 0.196 |
| Breed*Sex | 2 | 0.079 | 0.062 | **0.009** | **0.038** | 0.085 | 0.283 | 0.309 |
| Age*Sex | 8 | 0.897 | 0.950 | 0.498 | 0.868 | 0.804 | 0.935 | 0.988 |
| Breed*Age*Sex | 11 | 0.240 | 0.563 | 0.083 | 0.151 | **0.024** | 0.095 | 0.121 |

Note: FO: Following; SR: Stranger reaction; GR: Gunshot response; ST: stability test; FT: Fetch test; TW: Tug of war; AI: Attention item.

**Supplementary Table 4:** Estimated effect for each factor estimated from GLM.

| Factor | Level | FO | SR | GR | ST | FT | TW | AI |
| --- | --- | --- | --- | --- | --- | --- | --- | --- |
| Intercept |  | 3.68 | 3.65 | 3.28 | 3.45 | 2.73 | 3.16 | 3.60 |
| Breed | CKD | -0.27 | -0.20 | -0.55 | -0.35 | -0.89 | -0.90 | -0.72 |
|  | EGSD | 0.52 | 0.46 | 0.64 | 0.57 | 0.52 | 0.03 | 0.19 |
|  | GSD | 0.02 | -0.03 | 0.50 | 0.18 | -0.85 | -0.72 | -0.48 |
| Age | 2 | -0.13 | -0.19 | 0.11 | -0.03 | 0.08 | 0.05 | -0.15 |
|  | 3 | -0.63 | -0.65 | -0.36 | -0.49 | -0.19 | -0.34 | -0.43 |
|  | 4 | -0.42 | -0.48 | -0.28 | -0.40 | -0.41 | -0.32 | -0.45 |
|  | 5 | -0.24 | -0.22 | 0.02 | -0.13 | 0.13 | -0.05 | -0.19 |
|  | 6 | -0.48 | -0.52 | -0.11 | -0.31 | 0.27 | 0.13 | -0.13 |
|  | 7 | -0.37 | -0.37 | 0.03 | -0.12 | 0.09 | 0.00 | -0.10 |
|  | 8 | -0.22 | -0.34 | 0.18 | -0.09 | -0.08 | -0.26 | -0.31 |
|  | 9 | -0.62 | -0.62 | 0.37 | -0.17 | -0.72 | -0.55 | -0.51 |
|  | 10 | -0.68 | -0.53 | -0.16 | -0.33 | -0.48 | -0.66 | -0.85 |
| Sex | Sire | 0.73 | 0.69 | 0.86 | 0.81 | 1.41 | 1.12 | 0.89 |

Note: CKD: Chinese Kunming dog; EGSD: Eastern German Shepherd dog; GSD: German Shepherd dog; FO: Following; SR: Stranger reaction; GR: Gunshot response; ST: stability test; FT: Fetch test; TW: Tug of war; AI: Attention item.

**Supplementary Table 5:** Euclidean distance of estimated breed effect on behavioral stereotype among breeds.

|  | BMD | CKD | EGSD |
| --- | --- | --- | --- |
| CKD | 1.630460 |  |  |
| EGSD | 1.234463 | 2.646375 |  |
| GSD | 1.324764 | 1.260159 | 1.884171 |

Note: BMD: Belgium Malinois dog; CKD: Chinese Kunming dog; EGSD: Eastern German Shepherd dog; GSD: German Shepherd dog.

**Supplementary References**

Littell RC, Henry P, Ammerman CB, 1998. Statistical analysis of repeated measures data using SAS procedures. *Journal of animal science* **76**: 1216-1231.

Ruefenacht S, Gebhardt-Henrich S, Miyake T, Gaillard C, 2002. A behaviour test on German Shepherd dogs: heritability of seven different traits. *Applied Animal Behaviour Science* **79**: 113-132.
